# Expression of Placental Members of the Human Growth Hormone Gene Family Is Increased in Response to Sequential Inhibition of DNA Methylation and Histone Deacetylation

**DOI:** 10.1089/biores.2015.0036

**Published:** 2015-11-01

**Authors:** Esha Ganguly, Margaret E. Bock, Peter A. Cattini

**Affiliations:** Department of Physiology and Pathophysiology, University of Manitoba, Winnipeg, Canada.

**Keywords:** cell culture, gene expression, gene regulation

## Abstract

The genes coding for human (*h*) chorionic somatomammotropin (CS), *hCS-A* and *hCS-B*, and placental growth hormone (GH-V), *hGH-V*, are located at a single locus on chromosome 17. Efficient expression of these placental genes has been linked to local regulatory (5′ P and 3′ enhancer) sequences and a remote locus control region (LCR), in part, through gene transfer in placental and nonplacental tumor cells. However, low levels of endogenous *h*CS/GH-V transcripts are reported in the same cells compared with term placenta, suggesting that chromatin structure, or regulatory region accessibility, versus transcription factor availability contributes to the relatively low levels. To assess individual *h*CS-A, CS-B, and GH-V gene expression in placental and nonplacental tumor cells and the effect of increasing chromatin accessibility by inhibiting DNA methylation and histone deacetylation using 5-aza-2′-deoxycytidine (azadC) and trichostatin A (TSA). Low levels of *h*CS-A, CS-B, and GH-V were detected in placental and nonplacental tumor cells compared with term placenta. A significant >5-fold increase in activity was seen in placental, but not nonplacental, cells transfected with hybrid *h*CS promoter luciferase genes containing 3′ enhancer sequences. Pretreatment of placental JEG-3 cells with azadC resulted in a >10-fold increase in *h*CS-A, CS-B, and GH-V RNA levels with TSA treatment compared with TSA treatment alone. This effect was specific as reversing the treatment regimen did not have the same effect. An assessment of hyperacetylated H3/H4 in JEG-3 cells treated with azadC and TSA versus TSA alone revealed significant increases consistent with a more open chromatin structure, including the *h*CS 3′ enhancer sequences and LCR. These observations suggest that accessibility of remote and local regulatory regions required for efficient placental *hGH/CS* expression can be restricted by DNA methylation and histone acetylation status. This includes restricting access of the *h*CS 3′ enhancer sequences to available placental enhancer transcription factors.

## Introduction

Eukaryotic gene expression is affected by changes in chromatin configuration through histone and DNA modifications.^1,2^ Activation of placental members of the human (*h*) growth hormone (GH) and chorionic somatomammotropin (CS) gene family during placental development is facilitated by histone H3 and H4 (H3/H4) hyperacetylation as well as distal regulatory elements and a remote locus control region (LCR) that possesses enhancer activity.^3–7^ An assessment of DNA methylation in the *h*GH/CS gene locus suggested that undermethylation may also play a role in *h*GH/CS gene expression in placental versus nonplacental leukocytes.^8^

The four placental GH/CS genes (*hGH-V*, *hCS-A*, *hCS-B*, and *hCS-L*) are located at a single locus on chromosome 17 with the pituitary GH gene (*hGH-N*); they share greater than 90% nucleotide sequence similarity.^9^ The *h*GH-V gene codes for placental GH, and both *hCS-A* and *hCS-B* independently code for the same polypeptide hormone, CS (or placental lactogen), while *hCS-L* is a pseudogene.^10–12^ Both *h*CS and *h*GH-V are synthesized and secreted by syncytiotrophoblasts of the placental villi.^11^ Expression of the *h*CS/GH genes in placental cells has been linked to multiple regulatory regions, including hypersensitive sites (HS) III–V located remotely upstream in the LCR, P sequences located distally upstream of all four *h*CS/GH-V genes, and enhancer (3′-Enh) sequences located downstream of each *h*CS gene.^7,13–18^ Expression of *h*CS is high during pregnancy (grams of protein/day in the latter stages), and it has been reported that the genes can be differentially expressed during pregnancy.^12,19^ Levels of *h*CS/GH-V correlate with placental development and mass during pregnancy, and these pregnancy-specific hormones may help to control insulin sensitivity in normal pregnancies through positive effects on pancreatic β-cell mass.^20–22^

Assessment of *h*CS/GH-V gene regulatory regions has depended largely on the availability and use of human placental JEG-3, JAR, and BeWo choriocarcinoma cell lines. These cells express the *h*CS/GH-V genes, but at much lower levels relative to human term placenta.^23,24^ This correlates with a more open chromatin configuration of the *h*CS/GH-V gene locus in term placenta based on nuclease sensitivity studies.^25^

Hyperacetylation of histones and demethylation of DNA are often linked to a more open chromatin configuration.^2^ This increases accessibility and recruitment of transcription factors, including RNA polymerase II, and as a consequence gene expression.^26^ The effect of histone deacetylase (HDAC) or DNA methyltransferase inhibition on GH/CS RNA levels has not been reported; however, HDAC inhibitor, trichostatin A (TSA), and the hypomethylating agent, 5-aza-2′-deoxycytidine (azadC), have been used to promote an open chromatin configuration and look at effects on gene expression in placental cells and explants.^27–31^ There is also evidence of ectopic production of *h*GH/CS transcripts in nonplacental tissues and tumor cell lines, including from the breast.^32–37^ However, the identity of the specific *h*GH/CS genes expressed and the basis for expression is not well understood.

In this study, we compare individual *h*CS-A, *h*CS-B, and *h*GH-V RNA levels in placental and nonplacental cell lines relative to term placenta. We also use azadC and TSA to explore the possibility that changes in DNA methylation and/or histone acetylation status can differentially affect *h*GH/CS gene expression in placental and nonplacental cells.

## Materials and Methods

### Human term placenta samples

Human term placenta (HTP) samples were obtained after approval of the Health Research Ethics Board at the University of Manitoba from women with a body–mass index (BMI) of 20–25 kg/m^2^. BMI was assessed based on the prepregnancy weight.

### Cell culture and treatment with inhibitors

Human placental BeWo, JAR, and JEG-3 cells, as well as mammary gland MCF-7, and T47D, cervical HeLa, endometrial HEC-1-A, and brain U-87 tumor cells were obtained from the American Type Culture Collection. Cells were grown in monolayer on 100-mm culture dishes in RPMI 1640 (JEG-3, JAR, BeWo) or DMEM (MCF-7, T47D, HEC-1-A, HeLa, U-87) supplemented with 10% (v/v) fetal bovine serum and antibiotics (10 IU/mL penicillin, 10 mg/mL streptomycin). Cells were incubated in humidified atmosphere of 95% air and 5% CO_2_. For nontreatment studies, cells were normally harvested when 80% confluent.

For DNA methylation and/or HDAC inhibition, cells (3 × 10^5^/35-mm plate) were grown for 24 h, then fed with medium containing 5–50 μM azadC or 10–100 nM TSA for 24 and 18 h, respectively, before harvesting. For sequential treatment, cells were fed the medium with either (1) 50 μM azadC for 24 h, then refed with medium containing 100 nM TSA for 18 h; or (2) 100 nM TSA for 18 h, and then refed with medium containing 50 μM azadC for 24 h. As controls, cells were treated with a corresponding dose of dimethyl sulfoxide (DMSO) vehicle for comparison in all cases, or for single treatments with 50 μM azadC or 100 nM TSA, for the corresponding period during the sequential treatment regimens.

### RNA analysis

Total RNA was extracted using the QIAshredder and RNeasy Plus Mini kit (Qiagen). For reverse transcriptase–polymerase chain reaction (RT-PCR), 1 μg of RNA per reaction was reverse transcribed by the QuantiTect Reverse Transcription kit according to the manufacturer's instructions (Qiagen). Minus RT reactions were also done to assess genomic DNA contamination. For PCR, 4% (HTP) or 40% (cell lines) of RT reaction mixture was used with specific primers (Table 1). Detection of a 250 bp product common to all five members of the *h*GH/CS family was assessed using specific primers spanning exon 3 and exon 4 (Table 1) at an annealing temperature of 47°C for 30 cycles, as described.^23^ The presence of unspliced *h*GH/CS transcript or genomic DNA is detected as a 341–343 bp PCR product^23^ by agarose gel electrophoresis and ethidium bromide staining.

**Table 1.** **Primers Used for qPCR (*h* = human)**

RNA	Primer sequence
*h*GH/CS	For: CAGAAGTATTCATTCCTGCA
	Rev: TTTGGATGCCTTCCTCTAG
*h*CS-A	For: GGCTTCTAGGTGCCCGAGTA
	Rev: GCACTGGAGTGGCACCTTCA
*h*CS-B	For: CAGCAAGTTTGACACAAACTCA
	Rev: AGAAGCCACAGCTACCCTCT
*h*GH-V	For: GTTTGAAAGAAGCCTATATCCTG
	Rev: TCACCCTGTTGGAAGGTGTT
*h*GAPDH	For: TTGATTTTGGAGGGATCTCGC
	Rev: GAGTCAACGGATTTGGTCGT
*h* β-glucuronidase	For: TTGATTTTGGAGGGATCTCGC
	Rev: GAGTCAACGGATTTGGTCGT

Identification and quantitation of individual *h*CS-A, *h*CS-B, and *h*GH-V RNAs were done by real-time RT-PCR (qPCR) using specific primers (Table 1).^7^ Minus RT reactions were also done using the same primers as a control for genomic DNA. Specific amplifications were identified with a single peak in the melting curve and routine verification of a single PCR product by agarose gel electrophoresis. Gene expression was calculated relative to that of human glyceraldehyde-3-dehydrogenase (GAPDH). For azadC and TSA treatments, β-glucuronidase was used for normalization as its expression is less sensitive to azadC and TSA treatments under the conditions tested.

### Gene transfer and the luciferase assay

Cells were plated (1.5 × 10^5^/35-mm well) and 24 h later transfected with hybrid luciferase (Luc) genes (1 μg) using *Trans*IT^®^ -2020 transfection reagent (Mirus). Plasmids containing hybrid *h*CS-A promoter (496 bp) and firefly luciferase (Luc) reporter genes without (CSp.Luc)^38^ or with upstream 263 bp P (263P.CSp.Luc)^38^ and downstream 241 bp *h*CS-B enhancer sequences (CSp.Luc.241Enh) were used. For CSp.Luc.241Enh, the *h*CS-B 241 bp enhancer fragment was amplified using SV40pCAT-CSB enhancer plasmid DNA as a template,^39^ using primers containing an AatII restriction enzyme site (CSB-Enh-Forward: AGCTGCGACGTCGTCTACATTTCAGCTCATCAACTTGG, and CSB-Enh-Reverse: AGCTCGGACGTCCAGCTGTGAACACATGGGTCTCATCT. The amplicon was digested with AatII and ligated into the AatII site of CSpLuc and downstream of the luciferase gene. The construct was confirmed by sequencing. Cells were cotransfected with 20 ng/well of a hybrid *Renilla* luciferase gene (RLuc) directed by a minimal thymidine kinase promoter (Promega Corp.). Cells were harvested 18 h post-transfection, and luciferase activity (Luc/RLuc) was measured after cell lysis using the Dual-Luciferase^®^ Reporter (DLR™) Assay System (Promega Corp.).

### Chromatin immunoprecipitation assay

Chromatin immunoprecipitation (ChIP) was done by Zymo Research Corp. Briefly, cells were formaldehyde cross-linked and sonicated (200–700 bp fragments), and ChIP assay (*n* = 3) was done on each chromatin sample (27 μg) with 5 μg of antibodies (anti-H3, Abcam, ab1719; anti-H3ac, Millipore, 06-599; anti-H4ac, Millipore, 06-598). Normal rabbit immunoglobulin (IgG) polyclonal antibodies were used as a control (#PP64B, Millipore). The relative abundance of regions of the hGH/CS locus in immunoprecipitated and input DNA was quantified by qPCR (Table 2). Values for both H3/H4 hyperacetylation and IgG were normalized to values for H3, and data for hyperacetylated H3/H4 binding events are presented relative to IgG control values.

**Table 2.** **Primers Used for ChIP-qPCR**

RNA	Primer sequence
HS V	For: TCCCTCGGACCAGAACAC
	Rev: CCCAGGTAAAAGCAGCATGT
HS IV	For: TTCTCAGGGTTTGGGACTGA
	Rev: TGGGAAGAAGTGGTGGACTC
HS III	For: CACTGATGAGCTTGGCGTCAC
	Rev: CCTGCCACTTCCGCTCTCCA
(CS) E_L,A,B_	For: TCATCTTTGCGGTCCCTAAC
	Rev: AGCCCTCACTCCCTGAGATT
P_A_	For: CTGGACTAGCACCCCAGACTCATCAT
	Rev: TCGATGACACCCCTCTTGGATCCTCC
CS-Ap	For: GAGGAGCTTCTAAATTATCCAC
	Rev: CAGTTCTCTCTCCCTGCTTGA
P_V_	For: TTCCTGAAACATTATCGGACC
	Rev: TTGGATCCTCACACAGGCGG
GH-Vp	For: AGTGGCCCCAGGCCTAAACA
	Rev: CCTTCTCTCTCGCTGCTTCT

### Statistical analysis

Studies were done in triplicate unless stated otherwise, with at least two determinations per sample. Statistical analysis was done using Prism^®^ software. For two-category comparisons, unpaired *t*-tests were used, and for multiple comparisons, one-way analysis of variance (ANOVA) was used with a *post hoc* Tukey–Kramer test as appropriate. A value of *p* < 0.05 is considered statistically significant and is represented in figures as follows: * or #, *p* < 0.05; ** or ##, *p* < 0.01; and *** or ###, *p* < 0.001.

## Results

### Detection of hCS/GH-V transcripts in placental and nonplacental tumor cells

**FIG. 1.**
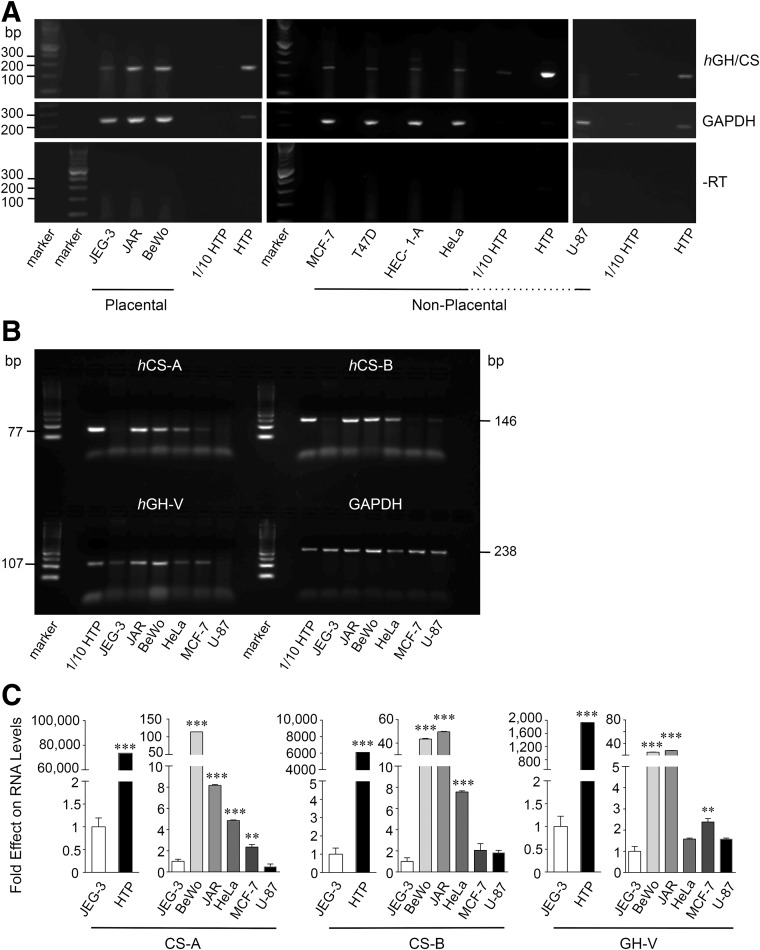
Assessment of total human (*h*)GH/CS RNA and relative levels of *h*CS-A, CS-B, and GH-V transcripts in placental and nonplacental tumor cell lines. **(A)** RNA was isolated from the tumor cells indicated as well as human term placenta (HTP) samples and assessed by reverse transcriptase–polymerase chain reaction (RT-PCR) using primers common to the five *h*GH/CS genes that span exon 3 and 4 sequences to yield a 250 bp amplicon (or 341–343 bp unspliced RNA or genomic DNA product), as well as glyceraldehyde-3-dehydrogenase (GAPDH). Minus RT reactions were also assessed. All products as well as 100 bp markers were visualized by agarose gel electrophoresis and ethidium bromide staining as shown in this representative image. **(B)** RNA was isolated from placental and nonplacental tumor cells and assessed by real-time RT-PCR (qPCR) using specific primers to *h*CS-A, *h*CS-B, and *h*GH-V, as well as GAPDH transcripts. All products as well as 50 bp markers were visualized by agarose gel electrophoresis and ethidium bromide staining as shown in this representative image. **(C)** Human CS-A, CS-B, and GH-V, as well as GAPDH transcripts were assessed as in **(B)**, and the latter was used to normalize the data. Values were calculated from a standard curve (absolute quantification). Results are expressed as fold difference in mean values relative to JEG-3 RNA levels, which has been arbitrarily set to 1.0, and error bars indicate standard error of the mean (*n* = 3). Comparisons of RNA levels between JEG-3 and HTP were assessed by unpaired two-tailed *t*-test and between cell lines by one-way analysis of variance (ANOVA) with post Tukey–Kramer Multiple Comparisons test (**p < 0.01, ***p < 0.001).

Total RNA was isolated from HTP as well as from placental BeWo, JAR, and JEG-3 tumor cells and nonplacental breast (MCF-7, T47D), uterine (Hec1A), cervical (HeLa), and brain/glial (U-87) tumor cell lines. Transcripts common to *h*GH/CS genes and combining exon 3 and 4 sequences, as well as human GAPDH RNA, were assessed by RT-PCR (Fig. 1A). Relative to HTP, the presence of low levels of *h*GH/CS transcripts was confirmed (Fig. 1A), with the exception of U-87 glioma cells where *h*GH/CS transcripts were not detected under equivalent conditions.

Individual *hCS-A*, *hCS-B*, and *hGH-V* expression was investigated in placental and nonplacental tumor cells by qPCR (Fig. 1B). For quantitation, values for *h*CS/GH-V transcripts were normalized to *h*GAPDH RNA levels and presented relative to JEG-3 cell levels, which are arbitrarily set to 1.0 (Fig. 1C). Expression of *h*CS/GH-V genes was detected in all cell lines, but this was a fraction of that observed in HTP based on a comparison with levels in JEG-3 cells. Relative levels of individual *h*CS/GH-V transcripts were all significantly greater in BeWo and JAR versus JEG-3 cells. However, a more variable pattern of relative expression was seen with JEG-3 when compared with nonplacental tumor cells; while *h*CS-A RNA levels were greater in HeLa and MCF-7 cells, *h*CS-B transcripts were greater only in HeLa cells and *h*GH-V transcripts were greater in MCF-7 cells alone (Fig. 1C). There was also no significant difference in individual *h*CS-A, *h*CS-B, and *h*GH-V RNA levels between JEG-3 and U-87 cells.

### The downstream CS enhancer region shows preferential activity in placental versus nonplacental tumor cells

**FIG. 2.**
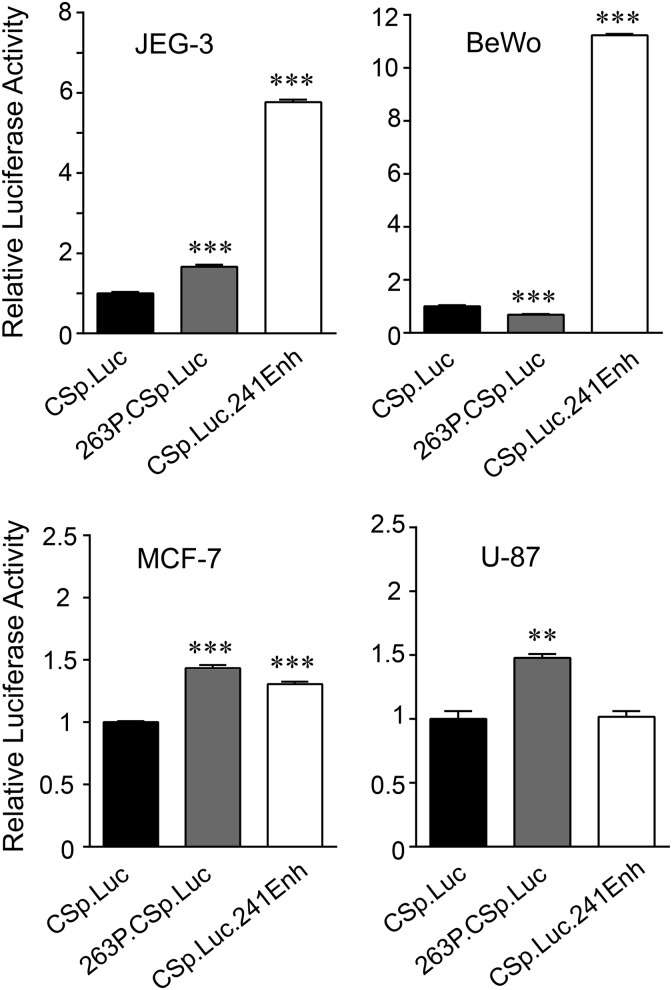
The downstream *h*CS enhancer region shows preferential activity in transfected placental versus nonplacental tumor cells. Hybrid *h*CS-A promoter (CSp)/luciferase (Luc) genes without (CSp.Luc) or with the 263 bp P sequence element (263P.CSp.Luc) or 241 bp *h*CS-B downstream enhancer element (CSp.Luc.241Enh) were used to transiently transfect placental (JEG-3, BeWo) and nonplacental (MCF-7, U-87) cells. To control for DNA uptake, cells were cotransfected with a hybrid *Renilla* luciferase gene (RLuc). Results are expressed as mean values (Luc/RLuc) relative to CSp.Luc activity, which was arbitrarily set to 1, and error bars represent standard error of the mean (*n* = 6). The mean values for CSp.Luc in JEG-3, BeWo, MCF-7, and U-87 were 1.895 ± 0.066, 0.788 ± 0.039, 3.110 ± 0.033, and 2.356 ± 0.148, respectively. Results for promoter activity were analyzed by one-way ANOVA with post Tukey–Kramer Multiple Comparisons test (**p < 0.01, ***p < 0.001).

Expression of *h*CS genes has been linked, in part, to local upstream P and downstream enhancer (3′-Enh) sequences and their associated factors.^7^ Activity of these regions and related availability of regulatory factors were assessed in transfected BeWo, JEG-3, MCF-7, and U-87 cells, using Luc reporter genes directed by a minimal *h*CS promoter with or without P or *h*CS-B 3′-Enh sequences (Fig. 2). There were modest (∼1.5-fold increase or decrease) effects of P sequences on *h*CS promoter activity in JEG-3, BeWo, MCF-7, and U-87 cells. A modest increase was also seen with 3′-Enh sequences in nonplacental MCF-7, but not U-87 cells. By contrast, a significant and more robust 5- to 12-fold increase in activity was observed with 3′-Enh sequences in JEG-3 and BeWo cells (Fig. 2). These data raise the possibility that placental enhancer factors are present in BeWo and JEG-3 cells, but unlike in the transfected Luc reporter gene, the *cis*-elements in the endogenous *h*CS genes are not accessible in the context of chromatin.

### Differential effects of DNA demethylase and HDAC inhibition on hCS/GH-V RNA levels

**FIG. 3.**
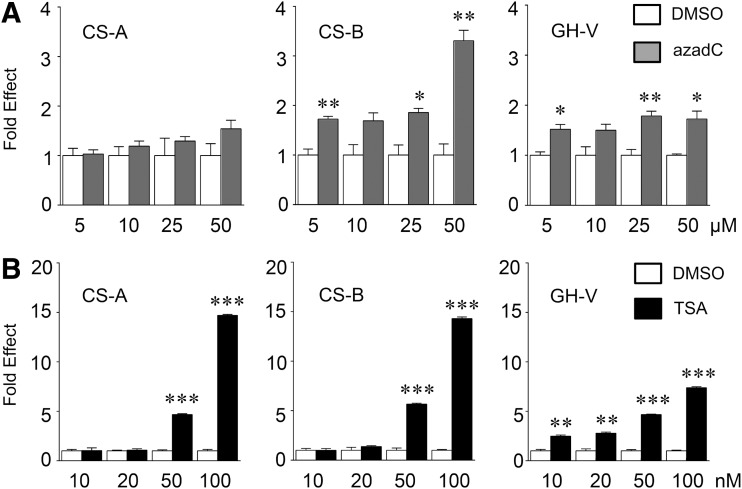
Differential effects of DNA demethylase and histone deacetylase (HDAC) inhibition on *h*CS-A, CS-B, and GH-V RNA levels. Placental JEG-3 cells were treated with increasing concentrations of the **(A)** DNA hypomethylating agent, 5-aza-2′-deoxycytidine (azadC), or vehicle (dimethyl sulfoxide [DMSO]) for 24 h or **(B)** HDAC inhibitor, trichostatin A (TSA), or DMSO for 18 h. RNA was assessed by qPCR using specific primers for *h*CS-A, CS-B, and GH-V, as well as glucuronidase transcripts, which were used to normalize the data. In all cases, mean values and standard error of the mean were determined, and results are expressed as fold effect of azadC or TSA treatment relative to independent control (DMSO) values, which are arbitrarily set to 1.0. Data were analyzed by *t*-test, and significant differences between DMSO control and azadC or TSA treatment groups are indicated by **p < 0.01 and ***p < 0.001 (*n* = 6).

To remodel chromatin and potentially increase transcription factor(s) accessibility, JEG-3 cells were treated with increasing concentrations of either azadC for 24 h or TSA for 18 h and levels of *h*CS/GH-V RNA assessed by qPCR (Fig. 3). For controls, cells were treated with DMSO vehicle under identical conditions for each treatment group, and RNA levels were normalized relative to β-glucuronidase transcripts. Treatment with 25 μM azadC or greater resulted in modest but significant and consistent increases in *h*CS-B and *h*GH-V, but not *h*CS-A, RNA levels in JEG-3 cells (Fig. 3A). Human GH-V transcripts showed greater sensitivity to TSA than *h*CS-A and B as levels were increased ∼2-fold with 10 and 20 nM TSA treatments. However, *h*GH-V as well as *h*CS-A and *h*CS-B RNA levels were all significantly increased ∼5-fold with 50 nM TSA, and a further increase (equivalent to 7- to 15-fold) was seen with 100 nM TSA treatment (Fig. 3B).

### Levels of hCS/GH-V transcripts are increased with sequential azadC and TSA treatments in JEG-3 cells

**FIG. 4.**
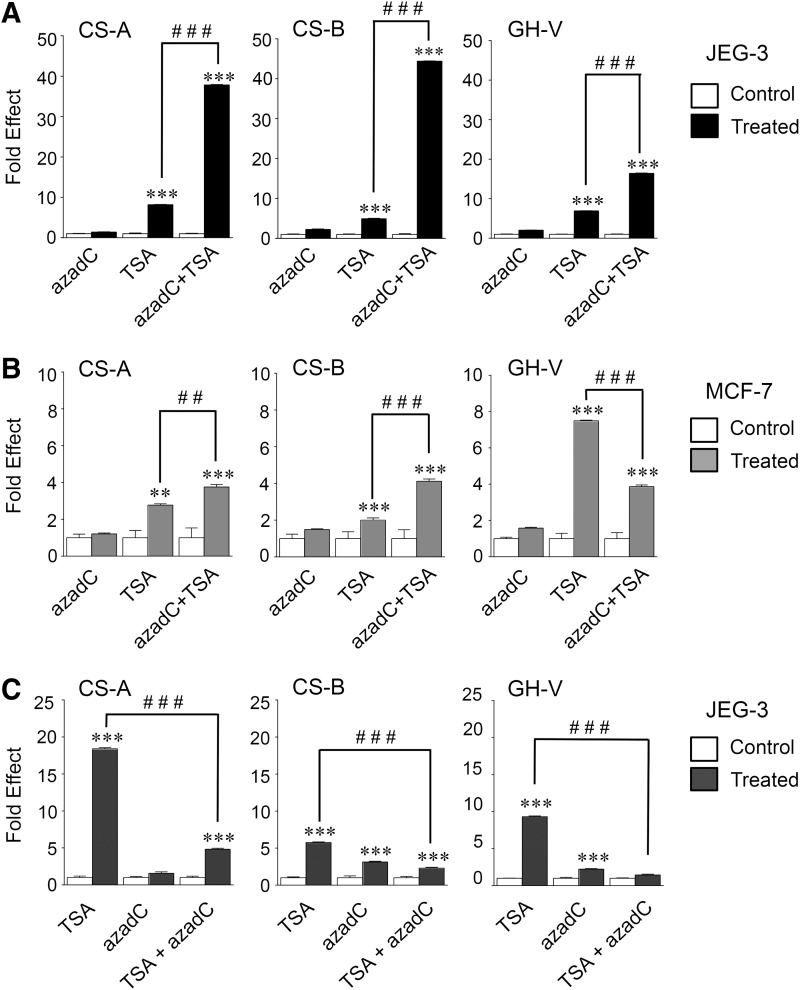
Human CS-A, CS-B, and GH-V RNA levels are increased specifically in placental JEG-3 cells with sequential azadC and TSA treatments. **(A)** JEG-3 cells and **(B)** nonplacental MCF-7 cells were treated with 50 μM 5-aza-2′-deoxycytidine (azadC) for 24 h; 100 nM TSA for 18 h; or azadC for 24 h, followed by TSA for 18 h (azadC+TSA). **(C)** JEG-3 cells were also treated with TSA for 18 h, followed by azadC for 24 h (TSA+azadC). In all studies **(A–C)**, cells were treated with vehicle (DMSO) for the corresponding length of time used for azadC and/or TSA (Control). Levels of *h*CS-A, CS-B, and GH-V RNA were assessed by qPCR as described in Figure 3. Mean values and standard error of the mean were determined, and results are expressed as fold effect of azadC, TSA, or azadC/TSA treatment relative to independent Control values for each treatment group/duration, which are arbitrarily set to 1.0 (*n* = 4–6). Data were analyzed by *t*-test. Significant differences between Control and azadC or TSA treatment groups are indicated by **p < 0.01 and ***p < 0.001. Significance between TSA and azadC/TSA treatment groups is indicated by ^##^*p* < 0.01 and ^###^*p* < 0.001.

The effect of sequential azadC and TSA treatments on *h*CS/GH-V transcripts in JEG-3 and MCF-7 cells was assessed by qPCR, given the individual effects of DNA demethylase and HDAC inhibition in JEG-3 cells. Significant increases in *h*CS-A (∼37-fold), *h*CS-B (∼44-fold), and *h*GH-V (∼15-fold) RNA levels were observed in JEG-3 cells treated with azadC and TSA versus TSA alone (Fig. 4A). By contrast, more modest (∼3-fold) increases in *h*CS-A and *h*CS-B were seen in MCF-7 cells (Fig. 4B), while *h*GH-V RNA levels were not increased, but rather decreased by ∼50% (Fig. 4B). To assess the specificity of the response, the study was repeated with reversal of the sequential treatment regimen (TSA, then azadC). By contrast to the effect of pretreatment with azadC, post-treatment with azadC mutes the stimulatory effects of TSA on *h*CS/GH-V transcripts (Fig. 4C).

**FIG. 5.**
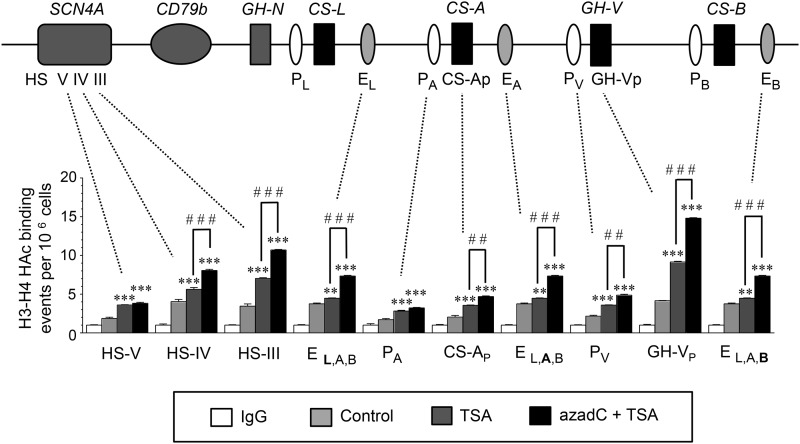
Histone H3/H4 hyperacetylation increases across the *h*GH/CS locus *in situ* with sequential azadC/TSA treatment of JEG-3 cells. ChIP assay was performed with antibodies specific to hyperacetylated (HAc) H3/H4 on chromatin isolated from JEG-3 cells treated without (Control) or with 100 nM TSA for 18 h, or 50 μM 5-aza-2′-deoxycytidine (azadC) for 24 h, then TSA for 18 h (azadC+TSA). Quantitative PCR was performed on both input and immunoprecipitated (bound) chromatin fractions (*n* = 3) with primer sets designed to detect regions of the *h*GH/CS locus control region (LCR) (hypersensitive sites [HS] III, IV, and V), downstream enhancer regions common to *h*CS-L, CS-A, and CS-B (E_L,A,B_), P sequences upstream of *h*CS-A (P_A_) and *h*GH-V (P_V_), and the proximal promoter regions for *h*CS-A (CS-Ap) and *h*GH-V (GH-Vp). Results are presented as HAc H3–H4 binding events relative to the immunoglobulin (IgG) control values for each primer set, which are arbitrarily set to 1. Values for HAc H3/H4 and IgG were all normalized to values for H3. Statistical analysis was performed compared with the control value for each primer set by one-way ANOVA with the post Tukey–Kramer Multiple Comparisons test (**p < 0.01 and ***p < 0.001). Comparison TSA and azadC+TSA for each primer pair was assessed by *t*-test; ^##^*p* < 0.01 and ^###^*p* < 0.001.

Based on the preferential response of the *h*CS 3′-Enh sequences in JEG-3 cells (Fig. 2), their H3/H4 hyperacetylation status was assessed in JEG-3 cell chromatin in response to sequential azadC and TSA versus TSA treatments by ChIP assay (Fig. 5). Primers were selected to assess the *h*CS/GH-V P and promoter sequences, HS III, HS IV, and HS V from the *h*GH/CS LCR, as well as (combined) *h*CS_A,B,L_ 3′-Enh, regions (Table 2). A trend toward increased H3/H4 hyperacetylation of the *h*GH/CS gene locus was suggested with TSA as well as sequential azadC and TSA treatments. However, significant increases in hyperacetylated H3/H4 in the *h*CS_A,B,L_ 3′-Enh sequences and the *h*CS-A and *h*GH-V promoters were observed with sequential treatments (Fig. 5). Increased histone hyperacetylation was also detected for the HS III and IV, but not HS V, regions of the *h*GH/CS LCR.

## Discussion

Relatively low levels of *h*GH/CS transcripts were observed in three placental and four of five nonplacental tumor cell lines as assessed by RT-PCR. Our detection of low levels of *h*GH/CS gene expression is consistent with previous studies.^23,24,32,40^ However, assessment was extended here to include individual *h*CS-A, *h*CS-B, and *h*GH-V RNAs in placental versus nonplacental tumor cells by qPCR. The results support low levels of expression in tumor cells versus HTP and are also consistent with BeWo and JAR cells displaying preferential placental expression of individual *h*CS/GH-V genes when compared with nonplacental HeLa, MCF-7, and U-87 cells.^23,24,32^

By contrast, the more similar levels of *h*CS and *h*GH-V transcripts in placental JEG-3 and nonplacental cells, including MCF-7 cells, would without further analysis suggest that JEG-3 cells do not support a mechanism for preferential placental gene expression compared with either BeWo or JAR cells. However, ectopic *h*CS expression is reported in MCF-7 cells^32^ and transfection of JEG-3 cells with *h*CS promoter/luciferase genes supports a mechanism for preferential placental cell expression. This included genes with and without regulatory P and 3′-Enh sequences that have been associated with placental *h*GH/CS gene expression.^3,14,15^ A differential response between placental and nonplacental cells was not observed with P sequences. However, a modest ∼1.5-fold increase and decrease in *h*CS promoter activity was seen in JEG-3 and BeWo cells, respectively. The significance of this difference is unclear, particularly as no effect or a slight decrease in the effect of P sequences on *h*CS promoter activity was reported previously in transfected JEG-3 cells.^16^ Thus, regardless of the direction of the response, these studies suggest the capacity of this region to respond to available transcription factors.

Preferential stimulation of *h*CS promoter activity was seen in JEG-3 and BeWo (but not MCF-7 and U-87) cells with inclusion of an *h*CS 3′-Enh element; this differential response was not observed with P sequences. Thus, this is consistent with the availability of *trans*-acting factors in placental tumor (but not nonplacental MCF-7) cells that can access the *h*CS 3′-Enh region, which presumably has a relatively open (chromatin) configuration in the context of transfected plasmid DNA, but cannot access the equivalent sequences in the endogenous hCS gene *loci*. A difference in the chromatin organization of the *h*CS/GH-V genes that affects accessibility of regulatory regions, including 3′-Enh sequences, could also contribute to the lower levels of expression detected in human choriocarcinoma cells versus HTP.

Changes in both DNA methylation and histone hyperacetylation status been linked to placental expression of the *h*GH/CS family.^3,8,41^ DNA hypomethylation and histone hyperacetylation are features of an open chromatin structure.^42–47^ Treatment with an inhibitor of histone deacetylation (TSA) resulted in an increase in *h*CS/GH-V RNA levels, but this was increased >10-fold when JEG-3 cells were treated first with a DNA hypomethylating agent (azadC) before inhibition of histone deacetylation. By contrast, a decrease in *h*GH-V and a more modest <2-fold overall stimulation in *h*CS RNA were seen with MCF-7 cells with sequential treatment. The specificity of the response in JEG-3 cells is indicated by the loss of this stimulation with prior azadC treatment when the treatment order was reversed and azadC is given post-TSA treatment. Although coordinated changes in DNA methylation and TSA-induced histone modifications have been reported for the luteinizing hormone receptor gene in JAR cells, TSA treatment was done in the continued presence of azadC; treatments were not sequential or reversed for comparison.^31^

The failure to see the same increases in *h*CS/GH-V RNA levels when TSA was used before treatment with azadC in JEG-3 cells suggests that specific (azadC-related) demethylation provides a preferred basis for efficient histone hyperacetylation and sequence availability. TSA decreases global DNA methylation, but in a manner distinct from azadC.^48^ Thus, the stimulatory effect on *h*CS/GH-V transcripts in JEG-3 cells is consistent with prior azadC treatment, enhancing the effect of TSA by interfering with the ability of methylcytosine-binding proteins to recruit HDACs to methylated regions.^2,49,50^

The specific increased *h*CS/GH-V gene expression with sequential treatment is associated with significant increases in hyperacetylation at *h*CS 3′-Enh sequences, the *h*CS-A/*h*GH-V promoters, as well as HS III and IV of the LCR. Multiple transcription factors have been linked to these regions through gene transfer and/or binding studies. Loss of C/EBPβ binding to 3′-Enh sequences correlates with a decreased *h*CS promoter activity with maternal obesity.^7^ DNA elements for FOXF1 in the proximal *h*GH-V promoter region and ETS-domain transcription factor family members in the HS III sequences have been linked to efficient activity.^4,51^ Furthermore, binding of AP-2 to HS III sequences correlates with H4 hyperacetylation in HTP,^4^ and CTCF sites are present within the LCR and gene locus.^52,53^ These observations do not rule out other regions and associated factors, rather they suggest that accessibility of remote and local regulatory regions linked to enhanced placental expression is restricted by DNA methylation and histone acetylation status in JEG-3 cells. This presumably contributes to the low levels of *h*CS/GH-V in choriocarcinoma versus HTP cells and comparable levels in nonplacental tumor cells. Based on our transfections with *h*CS 3′-Enh sequences, this occurs despite the availability of cognate *trans*-acting factors, including those required for preferential placental enhancer activity in human choriocarcinoma cells. This presumably contributes to the low relative levels of *h*CS/GH-V gene expression in JEG-3 cells and hence similarity to those detected in nonplacental tumor cells, where appropriate levels of regulatory factors are not available even when sequences were made accessible as with azadC and TSA treatments of MCF-7 cells.

## Abbreviations Used

ANOVA: analysis of variance
azadC: 5-aza-2′-deoxycytidine
BMI: body mass index
ChIP: chromatin immunoprecipitation
CS: chorionic somatomammotropin
DMSO: dimethyl sulfoxide
GAPDH: glyceraldehyde-3-dehydrogenase
GH: growth hormone
HDAC: histone deacetylase
HS: hypersensitive sites
HTP: human term placenta
IgG: immunoglobulin
LCR: locus control region
qPCR: real-time RT-PCR
RT-PCR: real-time reverse transcriptase–polymerase chain reaction
TSA: trichostatin A
